# Modelling airborne transmission of SARS-CoV-2 at a local scale

**DOI:** 10.1371/journal.pone.0273820

**Published:** 2022-08-30

**Authors:** Simon Rahn, Marion Gödel, Gerta Köster, Gesine Hofinger

**Affiliations:** 1 Department of Computer Science and Mathematics, Munich University of Applied Sciences HM, Munich, Germany; 2 Department of Informatics, Technical University of Munich, Garching, Germany; 3 Team Human Factors, Hofinger, Künzer & Mähler PartG, Ludwigsburg, Germany; VIT University, INDIA

## Abstract

The coronavirus disease (COVID-19) pandemic has changed our lives and still poses a challenge to science. Numerous studies have contributed to a better understanding of the pandemic. In particular, inhalation of aerosolised pathogens has been identified as essential for transmission. This information is crucial to slow the spread, but the individual likelihood of becoming infected in everyday situations remains uncertain. Mathematical models help estimate such risks. In this study, we propose how to model airborne transmission of SARS-CoV-2 at a local scale. In this regard, we combine microscopic crowd simulation with a new model for disease transmission. Inspired by compartmental models, we describe virtual persons as infectious or susceptible. Infectious persons exhale pathogens bound to persistent aerosols, whereas susceptible ones absorb pathogens when moving through an aerosol cloud left by the infectious person. The transmission depends on the pathogen load of the aerosol cloud, which changes over time. We propose a ‘high risk’ benchmark scenario to distinguish critical from non-critical situations. A parameter study of a queue shows that the new model is suitable to evaluate the risk of exposure qualitatively and, thus, enables scientists or decision-makers to better assess the spread of COVID-19 and similar diseases.

## Introduction

The outbreak of the coronavirus disease (COVID-19) started at the end of 2019. Within months, COVID-19 spread around the world and, ultimately, the World Health Organization [1] characterised the situation as a pandemic on 11 March 2020. It has affected many aspects of our daily lives. Therefore, scientists from various disciplines have attempted to answer urgent questions about the coronavirus and how it spreads.

From virology, we know that COVID-19 is caused by the severe acute respiratory syndrome coronavirus 2 (SARS-CoV-2). It is predominantly transmitted via respiratory fluids, making aerosols a likely route of transmission [2, 3].

Modelling plays an important role in epidemiology. Computer simulations help better understand the dynamics of a pandemic when ethical concerns prohibit experimental studies. In the following, we discuss several approaches whose scope ranges from large to small populations.

In their compartmental Susceptible-Infected-Removed (SIR) model, Kermack and McKendrick [4] describe the course of an epidemic, more precisely, the number of susceptible (S), infectious (I) and removed (R) individuals among a population, with ordinary differential equations. This model was modified, e. g. by adding an exposed state (SEIR) to simulate the latent period or by accounting for possible reinfection after temporal immunity (SIRS). The deterministic approach approximates a stochastic process of contact networks and, thus, is only valid for large populations. Kühn et al. [5] divide the population of Germany into its counties and several age groups. For each of these groups, they apply an extended SIR model and connect the groups by graphs to account for interactions. That is, this approach combines SIR-like and network models.

Network models also allow to estimate the dynamics of a disease. Müller et al. [6] simulate the spread in larger populations on the scale of regions or countries over several months. As shown in [7], network models can also operate on a local level such as single facilities and small populations for short periods up to two weeks. However, the network approach is relatively abstract.

In contrast, agent-based models from the field of crowd simulation capture the spread of diseases among smaller populations and allow to gain information about the transmission of pathogens between individuals. Each agent represents a virtual person. It possesses individual properties, such as a health condition. The transition from one state of health to another, e. g. from susceptible to infected, depends on predefined rules. These rules are often based on mutual distances. For example, virtual persons close to an infectious one become infected after a certain time (see [8–12]). Ronchi and Lovreglio [13] expand the concept of proximity to further contact types, e. g. physical contact, proximity within a certain radius and occupancy of the same room or building. The overall time spent in contact determines the risk of exposure. Duives et al. [14] carried out a preliminary study that, other than most agent-based approaches, measures a virtual person’s cumulative exposure independent of the proximity to an infectious person. Infectious persons shed viruses through aerosols, droplets and fomites, whereas susceptible ones absorb virions depending on the contamination at their position. The overall model takes several aspects of SARS-CoV-2 transmission into account, resulting in a complex model with many parameters, some of which are difficult to quantify and cannot be validated at this point. This reduces the predictive power of the model. A similar approach is presented in [15]. This work introduces a new model for human locomotion and transmission via aerosols, which dilute and spread over time. The overall probability of infection is defined as the ratio between the virtual persons that inhale more than a certain threshold of aerosols and the total number of persons that are present in the scenario at the same time as the infectious case. A parameter study sheds light on the impact of two factors on the degree of exposure: the occupancy level of the indoor environment and the ventilation rate. Repeated simulations yield distributions for the model output, but an exhaustive study has not been performed. Besides, this model does not capture the exposure when the infectious person leaves the scenario. Apart from the contributions [14, 15], most virus spread models combined with microscopic crowd simulation rely on the concepts of contact time and proximity. However, infection is possible without obvious proximity. Aerosolised pathogens can remain at a position where they were emitted after the infectious person has gone. Models that focus on proximity barely capture airborne transmission.

Even if an infectious person does not move, airflow may spread the pathogens and cause infections at distant places. Such transport mechanisms can be simulated with computational fluid dynamics (CFD) models. For example, Vuorinen et al. [16] simulate how the aerosols of a coughing person travel. Cortellessa et al. [17] assess the risk of exposure for short distances between two stationary persons. We refer to the work of Liu et al. [18] for a broader overview of contributions from the fluid dynamics community related to airborne SARS-CoV-2 transmission. They perform CFD simulations to investigate aerosol transport for a spreading event that happened in a restaurant in Guangzhou, China, during the early phase of the COVID-19 pandemic. The CFD study takes into account complex effects, e. g. turbulence and thermal effects. Despite the model’s high level of detail regarding fluid flow, several simplifications and assumptions are inevitable. For instance, persons sitting around a table and other heat sources are represented by hemispheres. Also, the ambient conditions are not exactly known. This lack of knowledge necessitates repeated simulations with different parameter sets. Employing such methods for a single, relatively simple problem is computationally expensive. This becomes even more demanding if CFD and crowd simulations are coupled, prohibiting scenarios with more than a few virtual persons. CFD simulations would require a large amount of information to model each pedestrian’s respiratory system. This also introduces boundary conditions that are constantly in motion. We are unaware of any CDF package that is able to deal with this complexity. Apart from that, the degree of detail between these two models does not match.

Another type of models assesses the infection risk for airborne transmission based on the assumption that pathogen-carrying aerosols are well-mixed within the considered space. Riley et al. [19] laid the foundation for this approach. Based on Wells [20] work, they formulated the Wells-Riley equation, which describes the probability of infection for a steady-state concentration of infectious particles. Infection risks can be studied at a local level, but proximity is irrelevant. As a consequence, the effect of measures such as physical distancing cannot be analysed in detail. This approach has been adopted extensively. Wells-Riley-like models are applied, e. g. in [21, 22]. Salmenjoki et al. [15] simulate two scenarios with both an agent-based model and the Wells-Riley formulation. They compare the results of both approaches and find that the agent-based model yields higher infection risks than the Wells-Riley model.

In summary, macroscopic compartmental models, agent-based models and CFD simulations allow analysing the spread of diseases on different scales. Macroscopic models consider the overall dynamics of an epidemic, whereas microscopic models focus on pathogen transmission between individuals. Gaining knowledge about the transmission on a local scale is of particular interest in the context of COVID-19. CFD simulations could be employed for complex problems, but they are not always feasible due to a lack of resources, for example, computational power. Small-scale proximity models neglect that pathogens may persist in aerosols, even if their source is no longer close. We wish to help bridge this gap and ask this question: How can we model airborne transmission of SARS-CoV-2 between individuals for everyday situations in which several individuals gather and possibly move around?

This question involves various disciplines, which all have their own terminology. We explain important terms in the Definitions section. To answer this research question, we introduce the methodology and our microscopic crowd model in the Methods section. In section Mathematical model, we formulate a mathematical model for pathogen transmission via aerosol clouds. We then computerise the model, couple it with crowd simulation and provide a parameter set to match SARS-CoV-2 transmission in the Computerised model section. In section Reference scenario, we propose a scenario to which an agent’s degree of exposure in any other situation can be compared. Section Verification and validation shows that the model is implemented correctly and that its results reflect empirical data. In the Application section, we simulate a situation that is relevant for everyday life: transmission between pedestrians waiting in a queue. Section Conclusion summarises and provides an outlook.

## Definitions

This work combines several fields of research, which may result in ambiguous terminology. We use the following definitions to avoid misunderstandings:

- **Aerosol:** The term aerosol is of key importance to this study. Generally, it describes solid or liquid particles of any size suspended in a gas [23]. In the context of this work, the particles are aqueous respiratory droplets in air.
- **Aerosol particles:** Aerosol particles are often differentiated depending on their aerodynamic diameter. Following this classification, particles above a given size are called droplets, whereas particles with a smaller diameter are referred to as aerosol particles. Conventionally, the threshold for this distinction is set to 5 μm. However, recent studies argue that 100 μm would be a better choice since particles with a size of 100 μm can linger in the air for more than 5 s and can be inhaled [24, 25]. In this contribution, we refer to aerosol particles as particles that remain airborne for several seconds to hours, and all larger particles that sediment faster are called droplets.
- **Airborne transmission:** The distinction between aerosol particles and droplets is also used in the context of transmission paths. It results in the definition of airborne transmission and droplet transmission, respectively. Depending on how aerosol particles and droplets are distinguished, this terminology can be misleading and contradictory, as criticised e. g. in [16, 26, 27]. For the sake of rigidity, we use airborne transmission for transmission via pathogen-laden aerosol particles that are carried by air.
- **Pathogen:** This study focuses on airborne transmission of SARS-CoV-2. We speak of (infectious) pathogens in general since the underlying model could also be applied to other infectious micro-organisms that are transmitted in a similar way as the coronavirus.
- **Agent:** In the pedestrian dynamics community, an agent is a virtual pedestrian, whereas microbiologists refer to infectious agents in the sense of micro-organisms, such as a virus. For clarification, we use (virtual) person and (infectious) particle or pathogen instead of the ambiguous term agent.
- **Physical distancing:** During the COVID-19 pandemic, people were obliged to keep a prescribed distance to others. This is called social or physical distancing. We prefer the term physical distancing in the context of this work, since social distancing may also refer to other aspects, e. g. reducing one’s social interactions.

## Methods and materials

We adopt the classical modelling approach to build a new model for disease transmission. That is, we translate real-world observations into a mathematical formulation. Then, we implement this model as an algorithm and generate a calibrated simulation programme. This creates a virtual world in which we test various scenarios against empirical data. Verification and validation are essential steps to ensure that the software contains very few errors and that it yields results consistent with empirical observations. We validate our model by re-enacting two superspreading events.

The transmission model is integrated into Vadere [28], an established framework for microscopic crowd simulation, which provides several locomotion models. In this contribution, we use the new sub-model for transmission in combination with the state-of-the-art Optimal Steps Model [29, 30], but it is designed such that it is compatible with any locomotion model. The Vadere source code for reproducing the numerical experiments is publicly available on GitLab (https://gitlab.lrz.de/vadere/vadere/, version b3be8e6a). The supporting information S1 Dataset. contains the configuration and the simulation output of each numerical experiment. Scripts for the evaluation of the results are also included.

## Mathematical model for transmission via inhalation

In this section, we model the transmission of SARS-CoV-2 among individuals via exhalation and inhalation. We develop our model specifically for COVID-19, but it can be transferred to other diseases that also spread through pathogens bound to aerosol particles, such as influenza [31]. As a first step, we operationalise real-world observations and derive a mathematical model.

### The virtual persons’ state of health

Inspired by the compartmental SEI model, we define a virtual person’s health as susceptible (S), exposed (E) and infectious (I). Susceptible persons represent healthy persons. They inhale pathogens and accumulate them. The number of accumulated pathogens describes their degree of exposure. Exposed persons retain the attributes of susceptible ones. Whether an exposed person becomes infected or not can be estimated employing a dose-response relationship. In contrast to the SEI model, the transition to the infectious state does not play a role in our contribution since the time scale of our simulations ranges from minutes to a few hours, which is significantly shorter than the latent period for SARS-CoV-2. Infectious persons emit pathogens via aerosols. For simplicity, we do not distinguish between symptomatic and asymptomatic cases.

Independent of the current infection status, each virtual person has a respiratory cycle of equally long periods of inhalation and exhalation. Pauses in between are neglected. Hence, we obtain the respiratory frequency *f* and the corresponding period *T* = *f*^−1^. During exhalation, an infectious person emits pathogen bound to aerosol clouds. Susceptible persons inhale a fraction of these pathogens if their current position is within the aerosol cloud.

### Emission of pathogen

In the case of COVID-19, infectious persons emit pathogen mainly through aqueous droplets expelled during breathing, but also, e. g. by speaking or coughing. These expiratory events vary in intensity and, thus, in particle numbers and sizes [26], which in turn alters how the aerosol particles spread through the air. Violent expiration causes also larger droplets, which follow a ballistic trajectory, whereas normal breathing produces mainly smaller aerosol particles [26] that remain in the air and form a cloud around the source. In either case, there is a suspension of liquid particles in the air, which is often called aerosol. There is no clear line between small and large particles [26]. In this contribution, we consider particles that stay airborne for at least several seconds. At this point, we focus on normal breathing. However, the concepts we present can be transferred to any kind of aerosol producing activity as long as the particles are small enough to stay airborne.

We are aware that particle clouds can be very complex. Literature offers two directions to address this problem: One assumes either a homogeneous distribution or an inhomogeneous distribution of aerosol particles within the considered space. The homogeneous case is justified for situations in which the air is well-mixed. These are typically indoors. Air mixing is often driven by thermal effects, e. g. body heat causes raising air around a person, and convective flow due to ventilation or movement. The Wells-Riley models are often based on this condition, whereas some extended versions of the Wells-Riley approach do not assume instantaneously well-mixed air. For instance, the model presented in [32] introduces a dilution ratio, which accounts for spatial and temporal differences of airborne contaminant concentrations between the positions of infectious and susceptible individuals. A more detailed resolution of the inhomogeneous case can in principle be obtained with CFD simulations. However, current publications with CFD simulations, e. g. [16–18], rely on strong assumptions and simplifications since considering human locomotion is out of scope in these studies. There is at least ground for doubt that the result of these simulations is more accurate than simpler approaches. Also, we argue that modelling such detail would be unsuitable for the degree of abstraction of the overall model. Besides, airflow is often turbulent, making its computation a major and still unsolved problem. Hence, we decided to keep the model simple and propose a compromise between the homogeneous and inhomogeneous assumption, similarly to Zhang and Lin’s work [32]. We model the distribution of aerosol particles resulting from one exhalation as temporally inhomogeneous and spatially homogeneous within a bounded area. The bounded area can extend over time.

**Fig 1:**
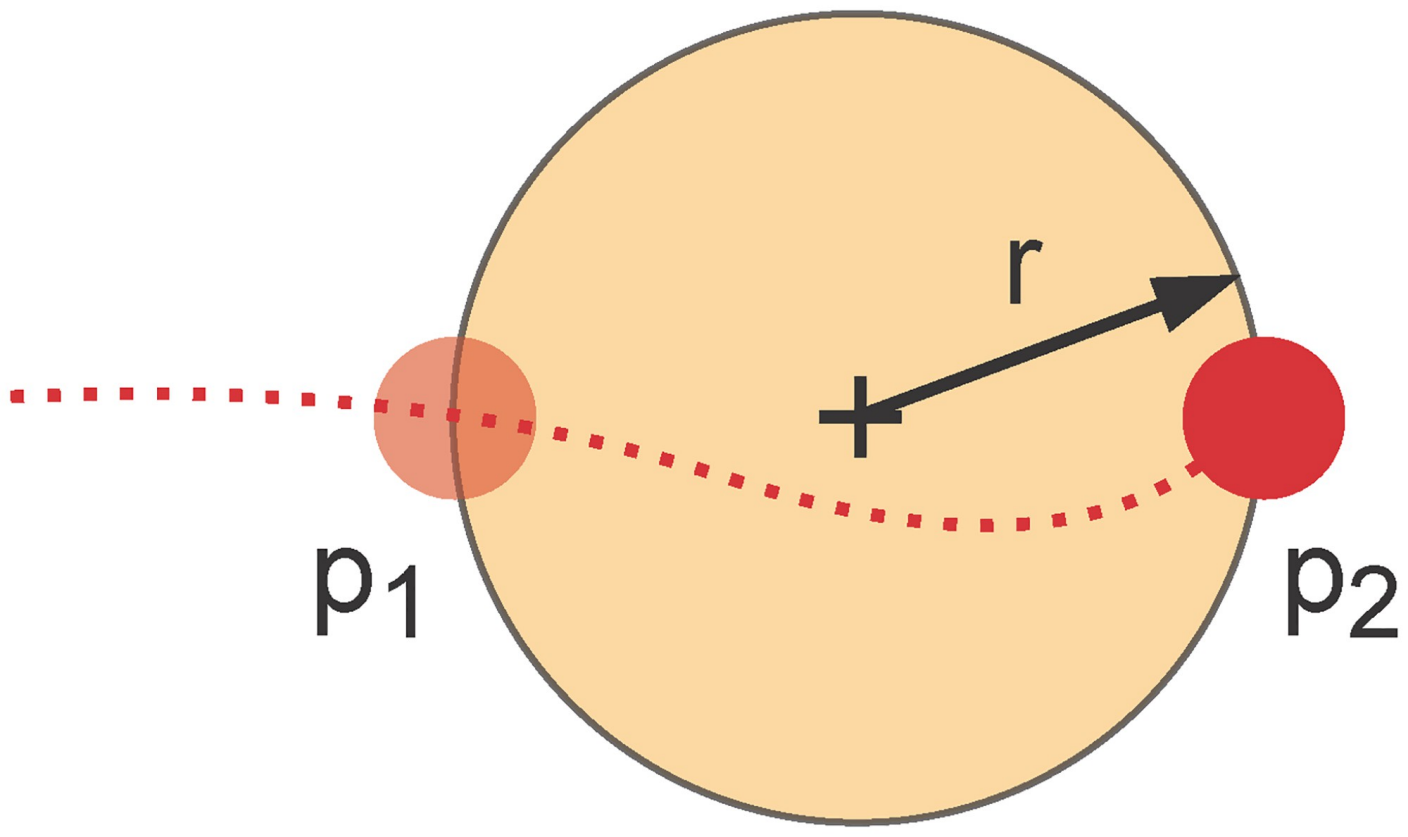
Infectious aerosol cloud. An infectious person (red) emits aerosol clouds (orange). The dotted line represents the person’s trajectory from left to right. It starts exhaling at *p*_1_ and stops at *p*_2_. The cloud with radius *r* coincides with the midpoint of line segment *p*_1_ to *p*_2_.

An infectious person creates an aerosol cloud containing pathogen particles with every exhalation (Fig 1). Since pedestrian dynamics are typically modelled in two dimensions, we represent each aerosol cloud by a circle in the horizontal layer. Kudryashova et al. [33] support this hypothesis. They divide the spread of aerosols into two phases: primary scatter and formation of a spherical cloud around the source, followed by diffusive propagation of aerosol particles. The latter cannot be explained by mere molecular diffusion but also by micro airflow, effectively resulting in greater diffusion coefficients. In our case, transport of aerosol particles is actually dispersive, predominantly driven by convection and only little influenced by diffusion. Molecular diffusion is negligible on our scale while information about convective airflow in our scenarios is unavailable. Therefore, we choose to focus on the simplest situation, with stagnant air, as can be found to some degree in confined spaces without ventilation. Such places are deemed particularly risky for SARS-CoV-2 transmission. Despite the lack of knowledge, we aim to describe the propagation of aerosol clouds very generally. Kudryashova et al. [33] conclude from experimental studies and mathematical modelling that aerosol particles suspended in still air would cover the area within the distance of one to two metres after 5 to 20 min. Moreover, the concentration reached a uniform distribution in approximately 3 to 5 min. We simplify this and assume instantaneous, spherical, and homogeneous scattering of aerosol particles. The sphere exhibits a volume of $V=\frac{4}{3}r^{3}\pi$. Of course, the two-dimensional model visualises only a circle with radius *r*, which represents the horizontal layer at the height of the virtual persons’ heads. The initial radius *r* is equal for all clouds. We let the cloud form over the entire period of exhalation *T*, so the shape is centred between the position, where the virtual person starts breathing out, *p*_1_, and the position, where it stops, *p*_2_. If the person walks faster, more precisely with speed |*v*| > 2*rT*^−1^, the distance between *p*_1_ and *p*_2_ is greater than the diameter of the circle. One could introduce more complex shapes to account for aerosol clouds that are stretched. We abandon this option because the exact shape of a single cloud has little influence on the simulation output as long as the pathogen concentration of this cloud does not suffice to infect other virtual persons.

We try to account for local air movement caused by walking persons. Any virtual person *i* who passes through a cloud with velocity *v*_*i*_ disperses aerosol particles. To capture the dispersion, we enlarge the clouds’ radius by Δ*r*(*t*) = Δ*t* ∑_*i*_|*v*_*i*_(*t*)| at each time step *t*. Here, the factor Δ*t* ensures that the model is independent of the length of the simulation step Δ*t*.

One can express the pathogen load of a cloud as homogeneous concentration *C*_*p*_ in particles per cubic metre. The concentration accumulates where clouds overlap. The initial pathogen load depends only on the infectiousness of the individual. It is therefore identical for all aerosol clouds emitted by the same virtual person. The pathogens of an aerosol cloud are inactivated after some time. In addition, aerosol particles evaporate, rise to higher air levels, or sediment. We simplify these complex processes by assuming an exponential decay of the number of pathogens with a half-life *T*_*a*_.

### Absorption of pathogen

While in the vicinity of one or more aerosol clouds, susceptible and exposed virtual persons breathe in pathogens. Each person absorbs the number of pathogen particles *N*_*p*_ contained in the tidal volume *V*_*T*_. The tidal volume is the volume inhaled and exhaled with each breath [34]. From this follows *N*_*p*_ = *C*_*p*_*V*_*T*_ (1 − *E*_*p*_). Masks reduce *N*_*p*_ by their effectiveness *E*_*p*_. *C*_*p*_ is the sum of the pathogen concentrations of all aerosol clouds at the virtual persons’ position. We neglect that inhalation removes pathogens from the surroundings.

The number of pathogens accumulated by a virtual person describes its degree of exposure. This degree of exposure can be translated into a probability of infection, typically through dose-response curves. We refer the interested reader to Haas et al. [35] for more details on quantitative microbial risk assessment. Brouwer et al. [36] identify important properties that should be considered when developing biologically plausible dose-response models. A very simple example is an exponential relationship. However, calibrating such models poses a challenge since, at present, important quantities such as the median infective dose of SARS-CoV-2 in humans is still uncertain. The infective dose probably depends on the individual, the variant of the virus and the route of inoculation. Karimzadeh et al. [37] estimate that more than 10^2^ viruses are required for infection. Popa et al. [38] analyse epidemiological clusters and infer that, on average, more than 10^3^ viral particles can successfully start an infection, but smaller quantities may suffice. Since there is no consensus on a dose-response model for SARS-CoV-2 and data for its calibration is scarce, we introduce the simplest model as a place holder: Persons who inhale more pathogens than a certain threshold are considered ‘high risk’. It can be exchanged when more consolidated knowledge becomes available.

## Computerised model

Here, we combine the transmission model with the Optimal Steps Model [29, 30], a state-of-the-art locomotion model for pedestrian dynamics implemented in the open-source framework Vadere [28]. Vadere is well-established for crowd simulations, which is why we adhere to its software architecture when we define important requirements for a new feature: The code should be compatible with existing structures, modular so that the transmission model can easily be enabled or disabled for different locomotion models and flexible to allow for adaptations by other developers.

### Embedding in Vadere

This section covers the embedding of the transmission model in Vadere. It gives insight into the software structure and how developers can enhance the model or implement additional features.

**Fig 2:**
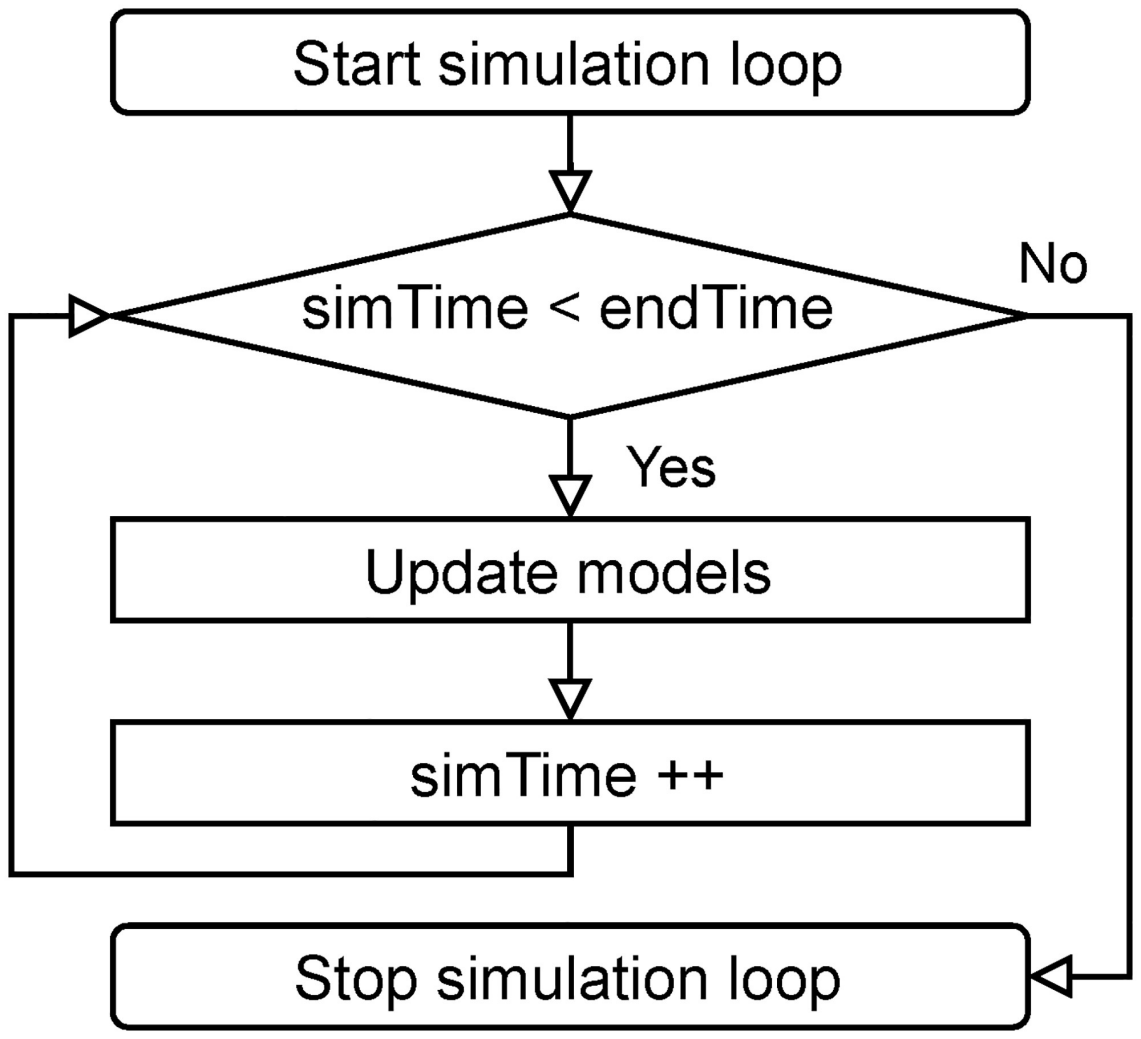
Simulation loop. Models are updated as long as the simulation loop is running.

The transmission model is integrated into Vadere’s simulation loop, a while-loop that updates all elements, mainly sources, targets and virtual persons’ positions, as long as the simulation is running (Fig 2). The simulation loop calls the transmission model independently of other models, keeping the programme modular and flexible. In particular, this allows combining transmission with any of Vadere’s locomotion model. Note that a user must select a locomotion model when running Vadere, whereas using the transmission model is optional.

**Fig 3:**
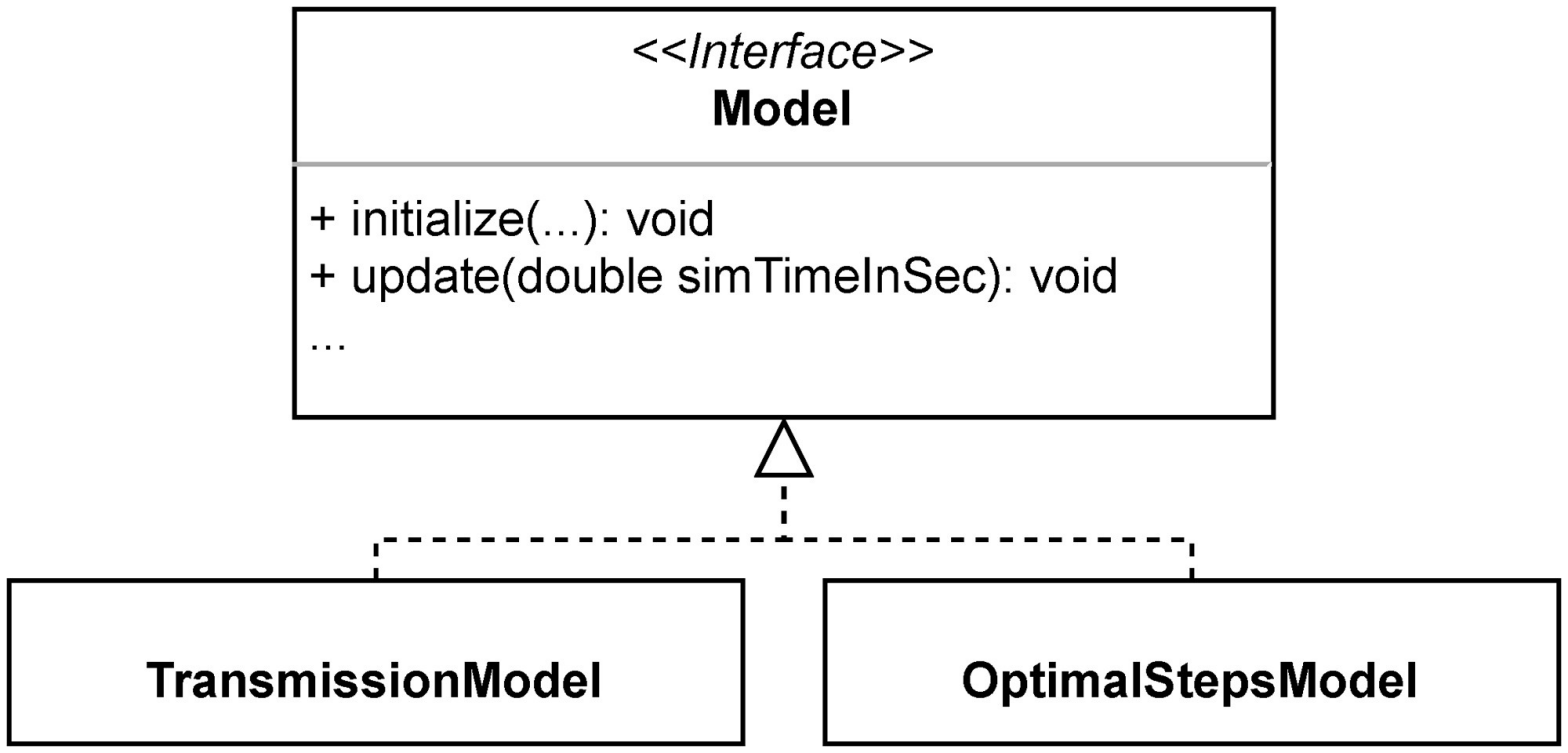
Class TransmissionModel. The class TransmissionModel contains the model core. Similar to the locomotion model OptimalStepsModel, it implements the interface Model.

Fig 3 visualises the structural embedding of the transmission model: The TransmissionModel implements the interface Model, as the locomotion models in Vadere do. Supplementary features or alternative models for disease transmission can be added on the same level.

TransmissionModel’s attributes contain general model parameters. Developers can adapt these attributes in the class AttributesTransmissionModel. We summarise the most important parameters in section Model parameters.

The methods of the class TransmissionModel contain most of the model’s logic. In essence, the method update(…) controls the emission of pathogen and updates aerosol clouds and the virtual persons’ health status. Each time an infectious person stops exhaling, an aerosol cloud is inserted into the topography, which can expand over time. Its pathogen load decreases exponentially. Aerosol clouds at the end of their lifetime are removed. Furthermore, the update method induces susceptible and exposed persons to absorb pathogens if they stand within aerosol clouds. The routine also updates each person’s HealthStatus, which contains absorbed pathogen load, respiratory cycle and infection status as its most important attributes.

Aerosol clouds are embedded as ScenarioElement and, thus, fit into the existing structure. Typical scenario elements are sources, targets or obstacles. This approach allows individual access and manipulation throughout the simulation runtime and facilitates the graphical representation of clouds.

### Model parameters

In this section, we summarise all important model parameters. Users can adapt the values directly in the input file. The parameters apply to all virtual persons and aerosol clouds within the same simulation. When a virtual person is spawned, the parameter values are assigned to the person’s attributes.

The virtual person’s health and aerosol clouds are partly defined by attributes listed in Table 1. The predefined values are explained in the next section. We are aware that, in reality, some parameters related to the health status are time-dependent or differ from individual to individual. However, we do not expect significant changes within the simulation time, which is very short compared to the period of communicability. We capture the heterogeneity among infectious persons, e. g. pathogen load or position, by running separate simulations for adapted parameter sets.

**Table 1:** Model parameters.

Parameter	Symbol	Value	Unit
pedestrianRespiratoryCyclePeriod	*T*	4	s
pedestrianPathogenEmissionCapacity	*N*	4	10^*N*^ particles
pedestrianPathogenAbsorptionRate	*R*	5 ⋅ 10^−4^	$\frac{{\text{m}}^{3}}{\text{inhalation}}$
aerosolCloudInitialArea	*A*_0_	7.1	m^2^
aerosolCloudHalfLife	*T*_*a*_	600	s

Model parameters describing a virtual person’s health status and aerosol clouds. The values correspond to a highly infectious person that exhales SARS-CoV-2.

### Calibration of a parameter set for SARS-CoV-2

The model presented here allows simulating any disease transmitted by aerosols. We select a set of parameters to match the transmission of SARS-CoV-2 through aerosol clouds formed by normal breathing. The parameters are physical in the sense that we can directly transfer them from the real into the virtual world. Their values are summarised in Table 1 and explained in the following.

An adult at rest breathes approximately 12 to 18 times per minute. We use an average of 15 breaths per minute, which implies a period of *T* = 4 s for each respiratory cycle. The exact values depend on factors such as the level of physical activity. However, slightly different breathing rates affect the quantity of absorbed pathogens significantly less than other parameters. The emission capacity in particular, which describes the number of emitted pathogens per breath, may vary in orders of magnitude for SARS-CoV-2. Ma et al. [39] found that some people exhale up to 4 ⋅ 10^5^ viral particles per minute. Assuming 15 breaths per minute and that the reported viral load refers to smaller respiratory particles, this means more than 10^4^ aerosolised viruses per exhalation. COVID-19 positive cases may emit significantly fewer pathogens, e. g. when viral replication is low for their variant [40] or because they are not at the peak of their infectiousness. Since we are interested in observing infection spread, we simulate a highly infectious person with 10^4^ virions per exhalation. To our knowledge, this represents the upper limit of a realistic range. Note that the related model parameter is logarithmised to base 10 in Table 1.

We now turn our attention to the absorbing, susceptible persons. The absorption rate *R* can be interpreted as the tidal volume *V*_*T*_ in cubic metres. For an adult, it is approximately 0.5 litre, that is 5 ⋅ 10^-4^ m^3^, per inhalation. If necessary, we can account for people wearing masks with filter efficiency *E*_*m*_. Rate *R* would decrease accordingly: *R* = *V*_*T*_ (1 − *E*_*m*_).

The aerosol clouds are characterised by the following parameters: the initial area and the half-life. Inspired by [33], we assume an initial radius of *r* = 1.5 m for circular aerosol clouds and, thus, an area of approximately *A*_0_ 7.1 m^2^. Hence, we obtain a volume of *V* 9.4 m^3^ and an initial pathogen concentration for each aerosol cloud of about 10^3^ pathogen particles per cubic metre.

We rely on reports from experience for the half-life of an aerosol cloud’s SARS-CoV-2 load. The half-life of artificially generated aerosols was found to last from 30 min to several hours [41, 42]. The exact value for the half-life *T*_*a*_ is not so important if the model output is interpreted qualitatively, as is the case in this contribution. However, it affects the dynamics of the model. A shorter half-life closely links exposure to the current location of an infectious person. On the other hand, a long half-life means that persons can become exposed even if the infectious case has left the area long ago.

## Reference scenario: Close contact

We gained parameter values on the transmission of SARS-CoV-2 from studies that have limitations. Therefore, we propose to evaluate simulated scenarios in relation to a reference: our benchmark scenario represents a so-called *close contact*, similarly to [14].

**Fig 4:**
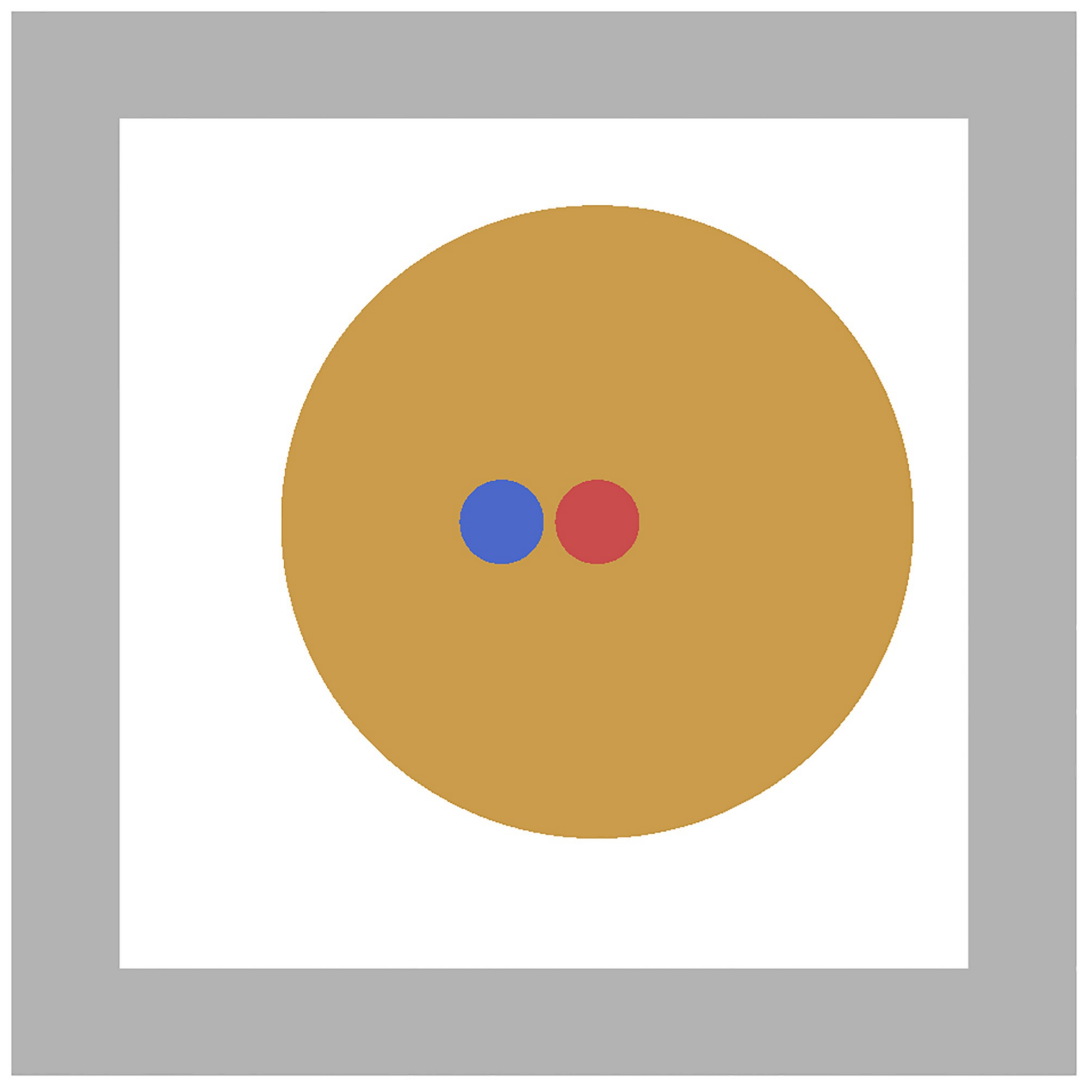
Close contact scenario. A highly infectious virtual person (red) emits pathogens bound to aerosols (orange) in an unventilated enclosed space. A susceptible person (blue) absorbs pathogens.

Governmental and institutional instructions regarding contact tracing during the COVID-19 pandemic define close contacts as follows: A susceptible individual and a confirmed case of COVID-19 occupy an enclosed space without adequate ventilation. They are in close proximity for a certain time so that the susceptible person inhales aerosolised SARS-CoV-2 particles. The leading scientific institute in Germany in the context of the COVID-19 pandemic, Robert Koch Institute [43], declares a distance of less than 1.5 m for more than 10 min as critical. The Centers for Disease Control and Prevention, U.S. Department of Health and Human Services [44], specifies 6 ft ≈ 1.8 m for more than 15 min. We follow the former definition with the parameter set from Table 1. Two virtual persons are placed less than 1.5 m apart (Fig 4). Both remain stationary for 10 min.

**Fig 5:**
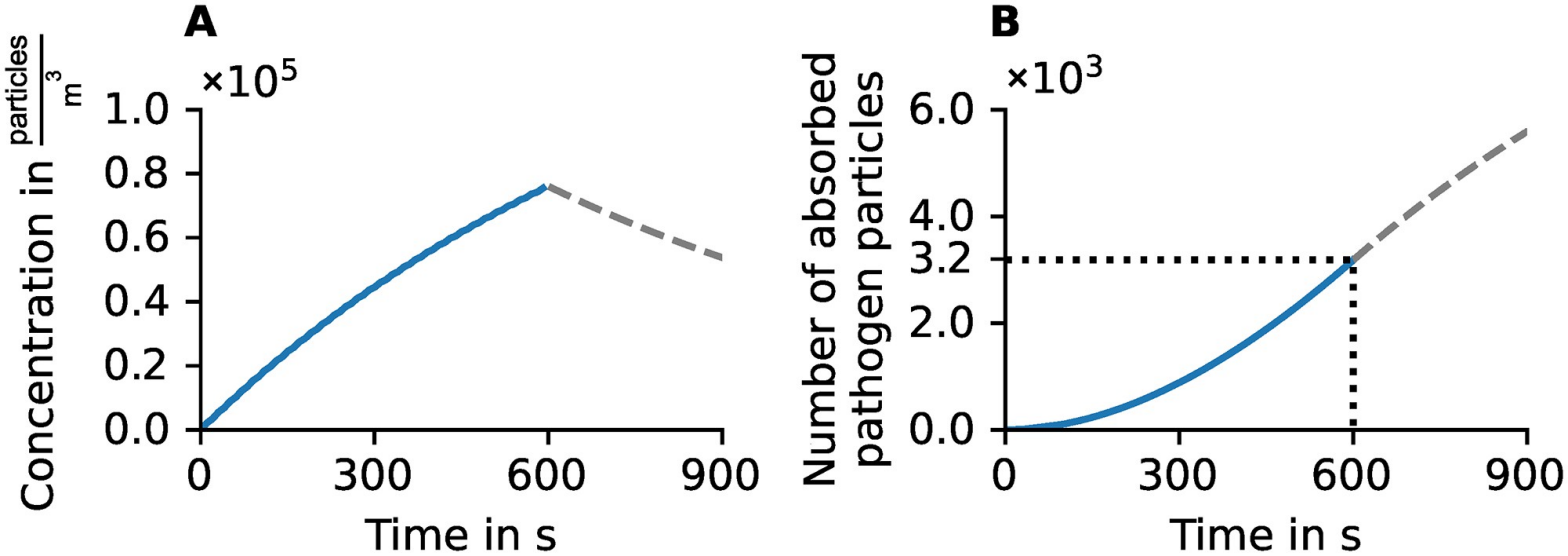
Pathogen concentration and absorption in the close contact scenario. An infectious person emits aerosol clouds so that the pathogen concentration builds up. The susceptible one absorbs approximately 3.2 ⋅ 10^3^ pathogen particles within the critical period of 600 s (dotted lines). The dashed lines represent an extension of the scenario: The infectious person leaves at *t* = 600 s, whereas the susceptible person remains and keeps absorbing pathogen from the exponentially decaying aerosol clouds. A: Pathogen concentration of aerosol clouds adds up. B: Pathogen load absorbed by the susceptible person.

The infectious person constantly emits aerosol clouds, thereby increasing the pathogen concentration (see Fig 5A). The susceptible one absorbs approximately 3.2 ⋅ 10^3^ pathogen particles within 10 min (see Fig 5B). In all further simulations, virtual persons who inhale a dose of *D* ≥ 3.2 ⋅ 10^3^ pathogens are considered a close contact, which we interpret as high risk of infection.

As soon as the infectious person leaves the scenario (*t* = 600 s), the pathogen concentration decreases exponentially. If the susceptible person remains, they will keep inhaling pathogen particles from the persistent aerosol clouds (see Fig 5).

## Verification and validation

Generally, careful verification and validation are necessary parts of the modelling and software development processes. We verify the transmission model by running unit tests. The test coverage of the core of our model reaches approximately 80%. Vadere’s continuous integration pipeline contributes additionally to detecting errors in the code, as every commit is tested automatically. The locomotion models have been verified with unit tests and validated with standardised scenarios according to the Guideline for Microscopic Evacuation Analysis (RiMEA) [28].

The validation of the transmission model, however, poses a challenge since empirical studies on local infection spread, in particular related to SARS-CoV-2, are scarce. Fortunately, some data on superspreading events are available and are sufficiently detailed to be compared to simulations. Superspreading means that one or relatively few individuals infect numerous people [45]. Since it also plays a significant role in the transmission dynamics of SARS-CoV-2 [46], it becomes the core of our validation.

Majra et al. [47] presented an overview of recorded events. Unfortunately, only a few situations can be simulated with our model, and even fewer are suitable for validation. Firstly, the model is designed to capture the transmission via aerosol clouds mostly stagnant for at least several minutes. Thus, strong flows should not dominate the air circulation at the event. Secondly, the event must not be too complex to be meaningfully represented in a simulation scenario. For example, the unknown routes of hundreds of guests at a carnival party would have to be guessed, making any comparison of data doubtful.

We find that the following events fit our purpose best: SARS-CoV-2 spread in a restaurant with ten infected people [48] and during a choir rehearsal, where 52 of 61 attendees became infected [2, 49]. Both events occurred and were investigated in the early phase of the pandemic when science and society were still relatively unaware of SARS-CoV-2. Consequently, other than the more recent events, measures such as physical distancing, air filtering or masks were absent. These would introduce further complexity because they must be adequately modelled and validated. We avoid this for the validation reported here. Effects of these measures can be introduced into the model by adapting the values of parameters, e. g. pathogen particles exhaled or half-life of the aerosol cloud. The reports provide information about the number of secondary cases, that is infected persons but not about the individual number of absorbed virions. We solve this by assuming that all virtual persons who reach the same level of exposure as the close contact in the reference scenario, that is 3.2 ⋅ 10^3^ virus particles, are considered high risk. We then compare the number of infected cases reported for the spreading event to the number of high risk persons we observe in the simulation. In addition, we also compare to a lower dose of 10^3^ virions. The parameter settings for the simulation seeds and the locomotion model for both validation cases are listed in the appendix (S1 and S2 Tables, respectively).

**Fig 6:**
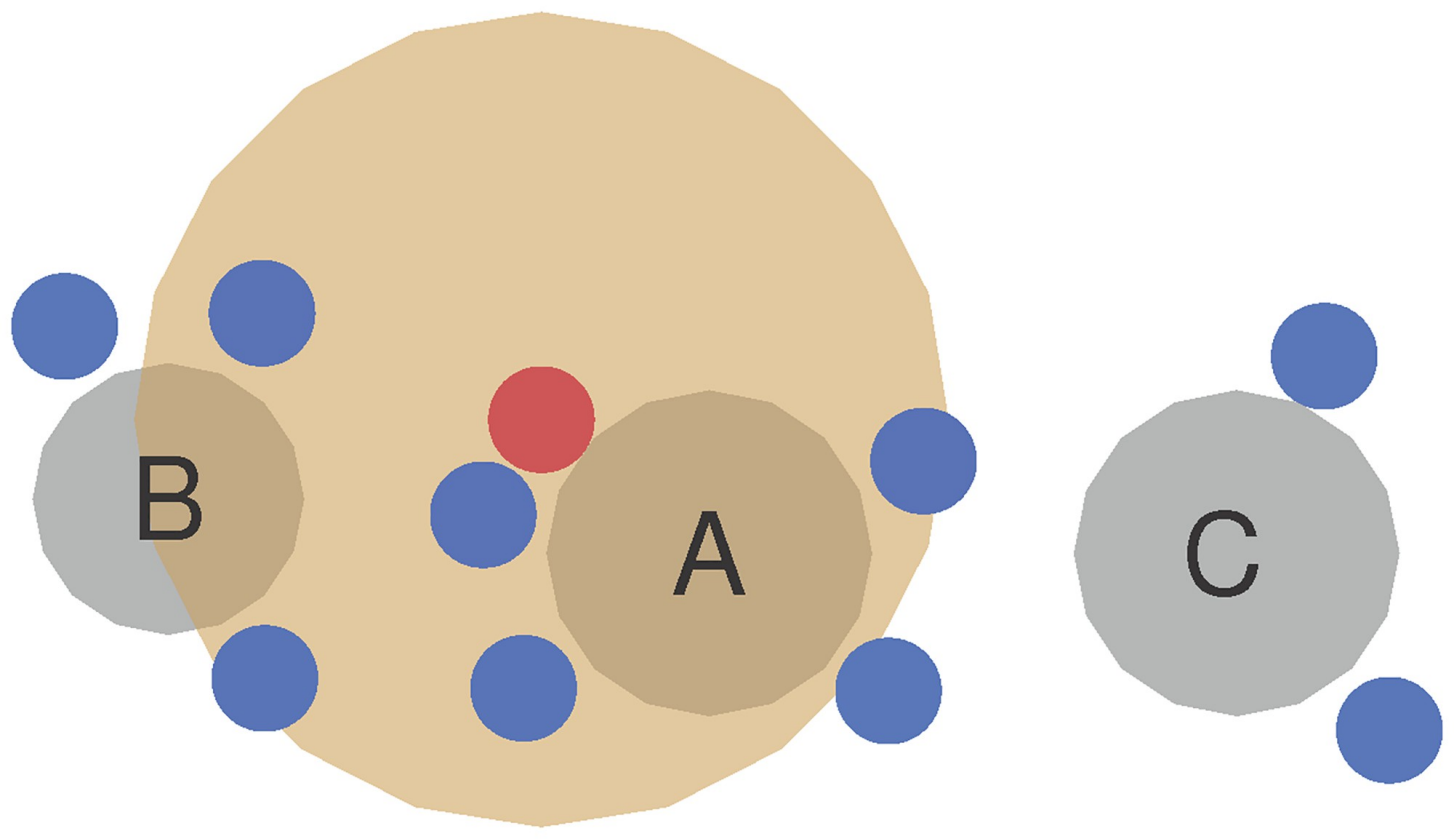
Restaurant scenario. Model of restaurant topography, including tables and seats, according to the seating chart in [48]. Susceptible (blue) persons sit in groups A, B and C around the tables (grey). The infectious (red) person emits aerosol clouds (orange).

We start with the spreading event in a restaurant in January 2020: Ten persons, divided into groups A, B and C, were sitting at adjacent tables. The infectious index patient belonged to group A. Group A shared the restaurant with group B for 53 min and with group C for 73 min. All ten individuals were tested positive after the restaurant visit. It was determined that the index case infected at least one member of groups B and C while in the restaurant. Further transmission among each group in the following days is considered possible. The topography is shown in Fig 6.

We consider our simulation as qualitatively valid if we observe infection spread below the numbers in the study. We argue that the simulation ignores airflow between tables from the restaurant’s ventilation as well as any transmission after the event. In the simulation, five persons become exposed, and among them, three from group A, two from group B and none from group C, that is, half the number of the true cases.

The choir rehearsal in March 2020 is significantly more complex: 1 of 61 attendees was symptomatic. After the practice, 33 people, including the index patient, were tested positive. Twenty further attendees are considered probable cases because they became ill but were not tested. One person, initially classified as a probable case, tested negative after the onset of symptoms. Thus, we use a minimum of 32 and a maximum of 52 secondary cases as reference values to which we compare our simulation results.

Again, we expect fewer high risk persons in our simulation than in reality. In addition, we do not have sufficient information about close interactions between the attendees during arrival and departure to include them in the simulation. As a consequence, we ignore opportunities for droplet transmission. Moreover, singing forcibly propels droplets, increasing pathogen spread.

**Fig 7:**
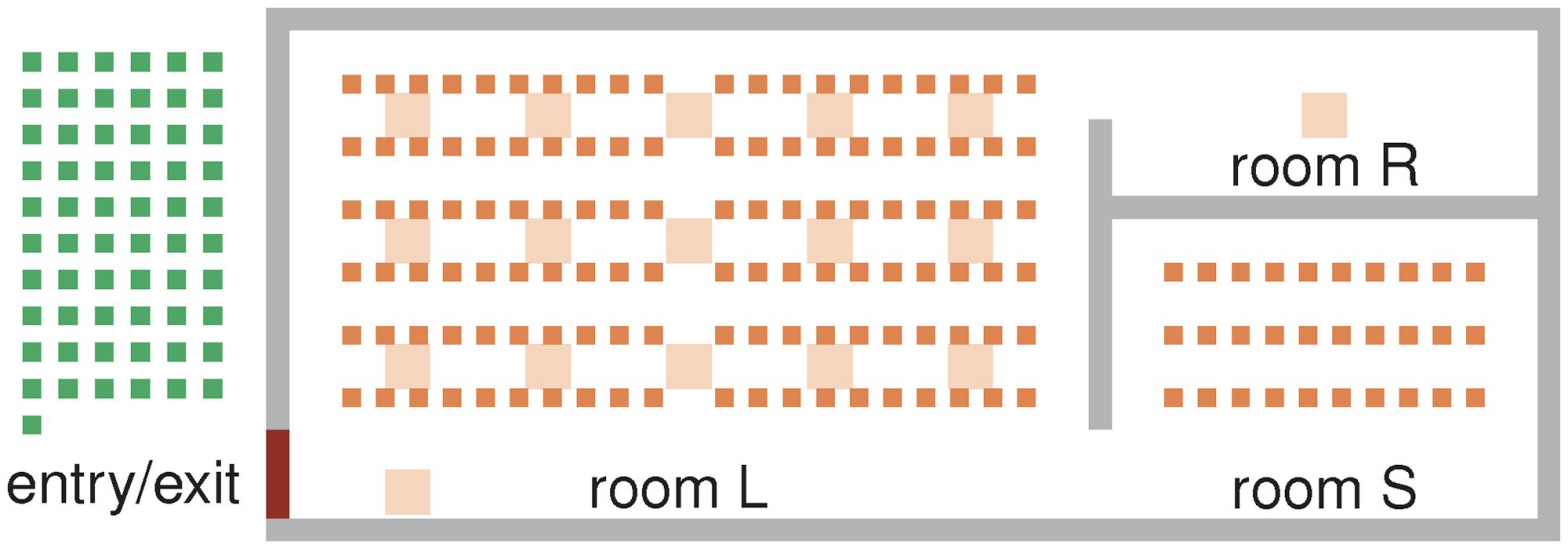
Topography of the choir practice scenario. A large (L) hall, a small (S) room and a restroom (R). Virtual persons are spawned by sources (green), they approach an intermediate target, remain there for a given period and move to the next one (targets all orange). Small orange squares represent chairs. Large, light orange squares define positions where virtual persons gather in small groups during the break.

The choir practice took place in a large room and, partly, in a smaller room. The attendees changed their positions from time to time, which we model by allowing the virtual persons to move from one intermediate target to another. The study did not provide a seating chart citing privacy concerns. Thus, we imitate the seating arrangement from [2] qualitatively but must make assumptions about the floor plan: Fig 7. The rooms cover an area of approximately 28 m × 12 m. Smaller intermediate targets (orange) mark possible seating positions during the practice sessions.

Miller et al. [2] report the following schedule: During the first *T*_1_ = 45 min, all choir members practised together in the large room L. Some seats between individuals were empty. A 45-min (*T*_2_) sectional rehearsal followed during which the attendees were divided into two groups, one in room L, the other in room S. The index case remained in room L. After that, there was a break of approximately *T*_3_ = 10 min. This allowed for mingling, and a few people, including the index case, used the restroom. The positions during the break are not reported. We assume that the choir members gathered in small groups distributed across room L. For the last session of *T*_4_ = 50 min, everybody returned to their original positions in room L.

We simplify the schedule. Firstly, we choose practice sessions of equal length (*T*_1_ = *T*_2_ = *T*_4_ = 45 min), which makes the definition of intermediate target positions in the simulation easier. Secondly, we ignore the fact that the attendees arranged chairs before and after the rehearsal, arguing that the time for this appears short compared to the entire practice. While these two aspects may be negligible, we acknowledge that the attendees’ exact positions in space and time would be essential. They would reveal where high pathogen concentrations can occur and where exposure is likely. Unfortunately, this information is not available and remains uncertain.

**Fig 8:**
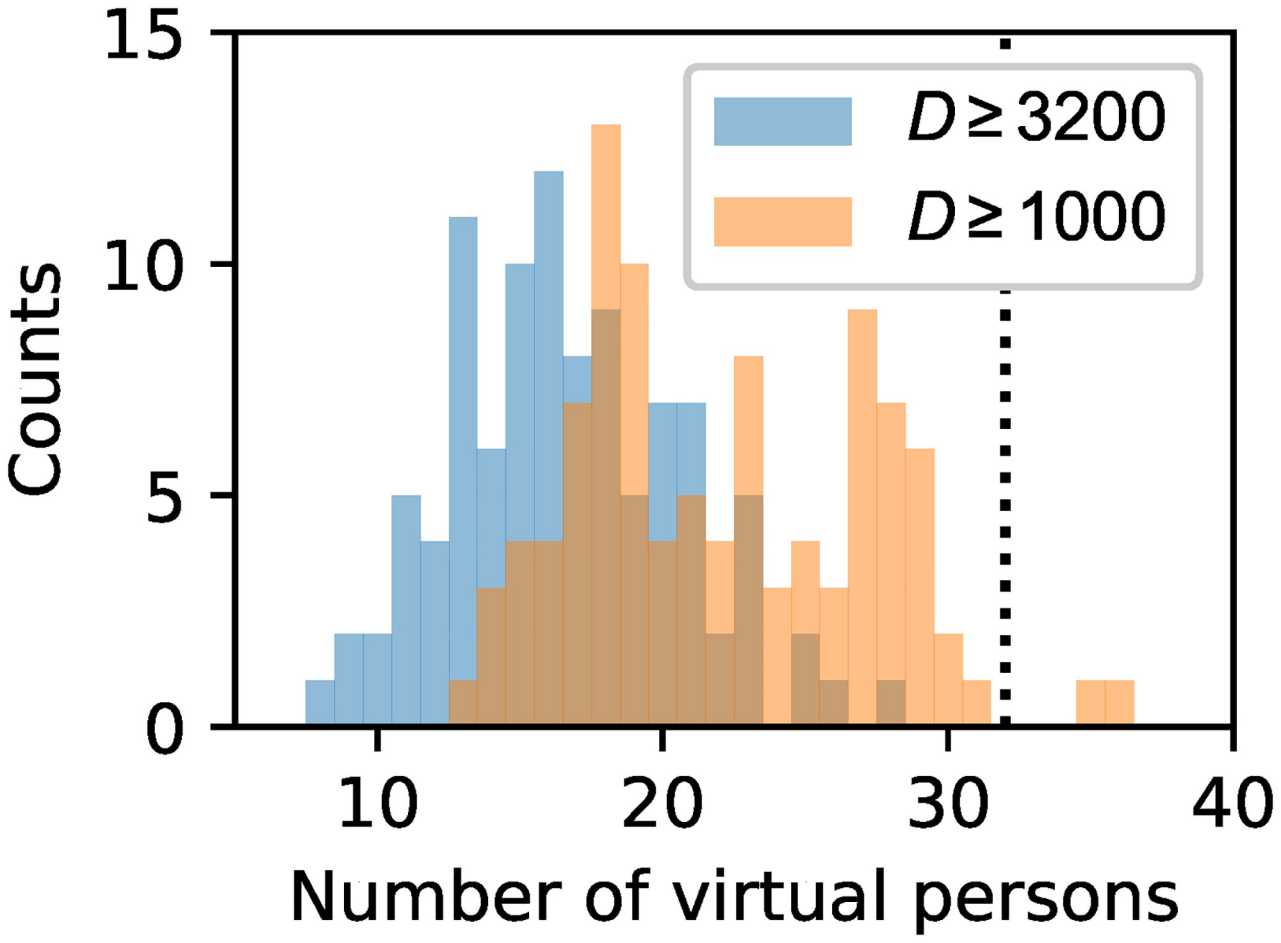
Virtual persons with degree of exposure *D* in the choir practice scenario. Number of persons for *M* = 100 simulations reaching a degree of exposure of *D* ≥ 3.2 ⋅ 10^3^ (close contact) and *D* ≥ 10^3^ virions. The dotted line indicates the 32 confirmed secondary cases of the true spreading event.

We deal with this by applying Monte Carlo techniques: We evaluate the model *M* = 100 times, collect the simulation output and summarise it statistically. The simulations differ only in the virtual persons’ paths. We achieve this by randomly mapping the intermediate targets to the individuals for each simulation set-up. Fig 8 shows a histogram of the number of high risk persons in the simulations.

We observe that the simulation results for both parameter choices are of the order of magnitude that was reported for the real event, but with fewer high risk persons. This is what we expected, and we argue that the simulation results support our model’s validity.

## Application to a queue scenario

In this section, we use the transmission model to evaluate exposure in a queue in front of a service unit, e. g. a counter at a shopping centre, a cinema, an office or any other similar situation.

### Numerical experiments

**Fig 9:**
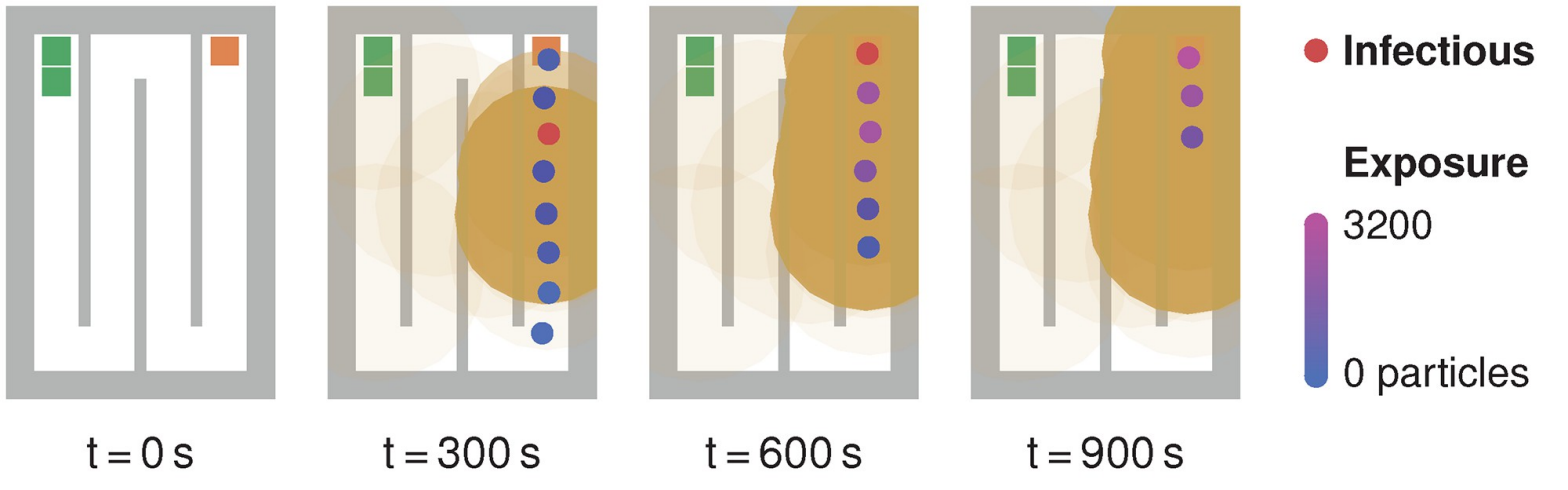
Queue scenario at different time steps. Susceptible persons (blue) are exposed to pathogens in aerosol clouds (orange circles) exhaled by an infectious person (red). The opacity of aerosol clouds reflects their current pathogen concentrations. The individuals’ colour indicates their degree of exposure.

We simulate one highly infectious person among several susceptible pedestrians (see Fig 9). In the green area, one source spawns nine susceptible persons, and a second source spawns a single infectious person at the fifth position. Belt barriers guide the meandering queue to the counter. After a fixed service time *T*_*s*_ = 120 s, each person immediately leaves the simulation. The topography represents part of a building, covering an area of 5 m × 7 m. The parameter settings for the simulation seeds and the locomotion model are listed in the supporting information (S1 and S2 Tables), allowing third parties to repeat and check our computer experiment.

Fig 9 shows the queue for several time steps. The colour, ranging from blue to violet, indicates the number of pathogens accumulated in a virtual person. All aerosol clouds remain at their initial positions because we assume no ventilation but only little air mixing caused by the pedestrians’ movement. As explained in detail in Mathematical model section, this assumption simplifies real fluid mechanics. CFD simulations could better capture the physics for stationary persons, unfortunately at high computational cost. In our simulation, high concentrations occur where many clouds are superimposed.

This pathogen concentration visualisation aids us in identifying potentially risky situations. The sixth, seventh and eighth positions in this queue, behind the infectious person, are critical, as persons directly step into and remain in the recently contaminated area. The persons at these positions absorb more than *D* = 3.2 ⋅ 10^3^ pathogen particles, that is, they carry at least the same infection risk as the close contact in the reference scenario. However, the seventh and eighth positions are not a close contact position because the infectious person is not closer than 1.5 m for more than 10 min. Can we reduce their degree of exposure by further increasing the distancing in the queue?

**Fig 10:**
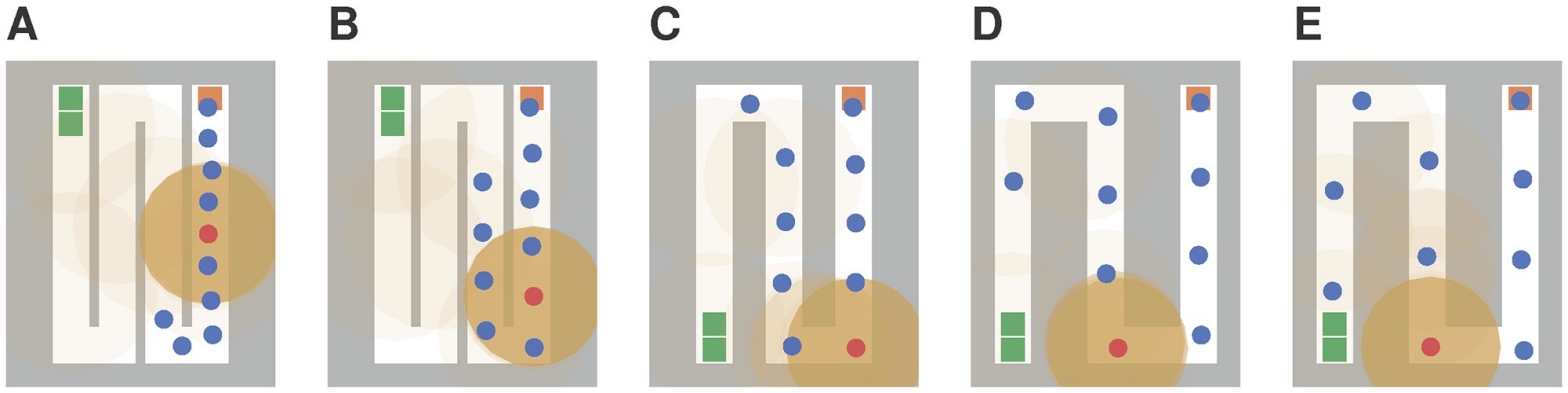
Queue scenarios with varying distances between virtual persons at *t* = 100 s. The scenarios A–E exhibit different distancing behaviours. This is achieved by increasing the potential parameter *p* and the centre-to-centre distance between the corridors *d*_*c*_. A: *p* = 0.5, *d*_*c*_ = 1.0 m; B: *p* = 1.0, *d*_*c*_ = 1.0 m; C: *p* = 1.5, *d*_*c*_ = 1.5 m; D: *p* = 2.0, *d*_*c*_ = 2.0 m; E: *p* = 2.5, *d*_*c*_ = 2.0 m.

**Fig 11:**
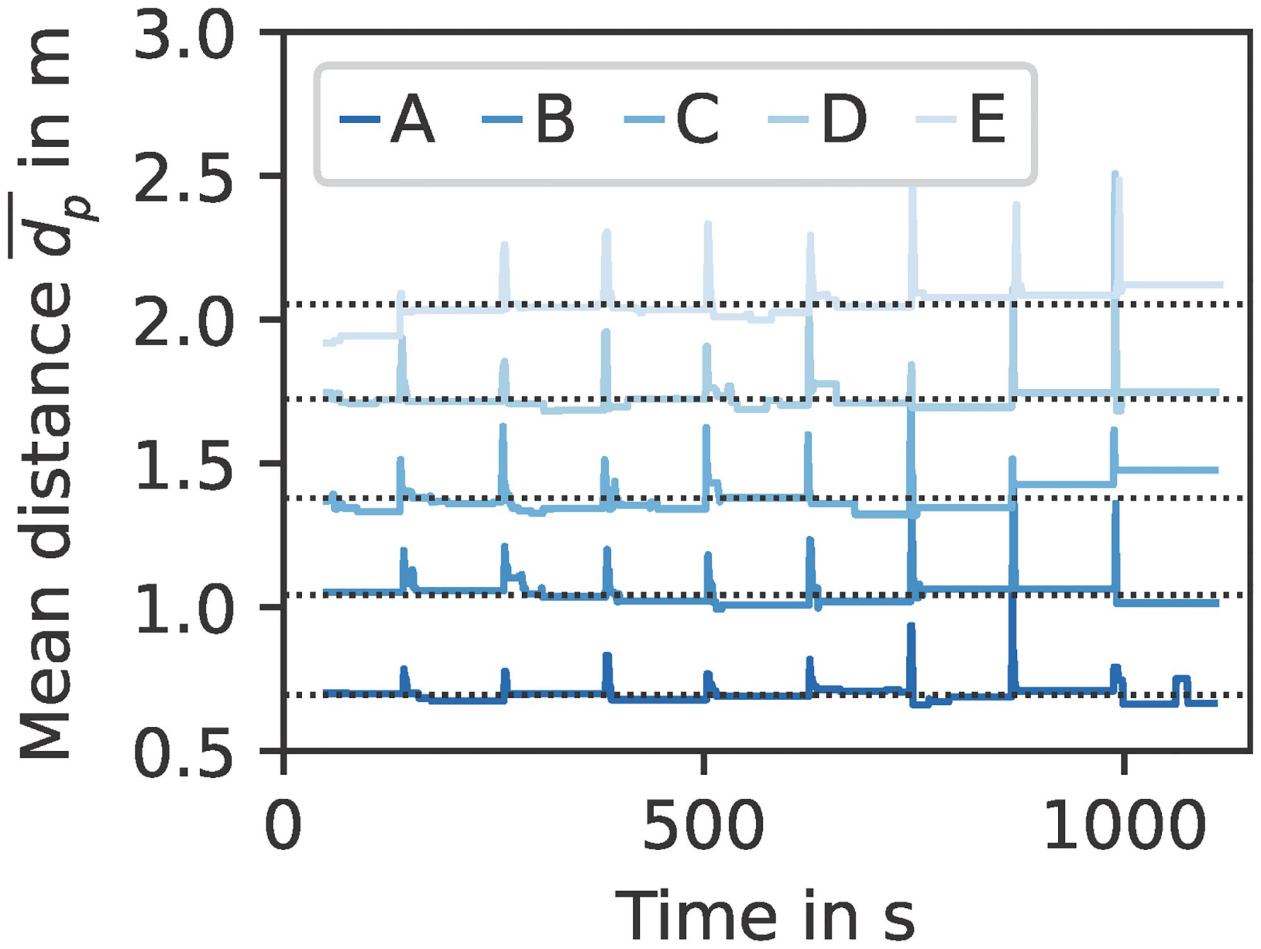
Distances between persons in the queue scenarios A–E over time. The mean centre-to-centre distance between queueing persons $\bar{d_{p}}$ increases with potential parameter *p* and corridor distance *d*_*c*_. We consider only the period *t* ≥ 50 s, that is when all persons have queued up. The peaks indicate time steps at which a person leaves the scenario and all others move up, leading temporarily to a greater mean distance. Dotted lines represent the time average of each series.

The locomotion model allows to simulate different distancing behaviours. Modelling physical distancing with the optimal steps model was demonstrated for a bottleneck scenario in [50]. However, we must adapt different parameters to obtain the right behaviour if we consider a queue under distancing regulations: The parameter pedPotentialPersonalSpaceWidth, in the following denoted by *p*, allows to change the extent of an individual’s personal space. We also have to modify the centre-to-centre distance of the corridors *d*_*c*_ to let the virtual persons keep mutual distances *d*_*p*_ ≥ 1 m. This represents, for instance, rummage tables that were used in shopping malls during the COVID-19 pandemic to guide customers towards the cash counter. Fig 10 shows the scenarios A–E with settings *p* = {0.5, 1.0, 1.5, 2.0, 2.5} and *d*_*c*_ = {1.0, 1.0, 1.5, 2.0, 2.5} metre. It should be noted that neither the potential parameter *p* nor the corridor distance *d*_*c*_ is equal to the actual distance between the persons *d*_*p*_. The actual distances can be evaluated as follows. We define $\bar{d_{p}}$ for each time step as the mean of the centre-to-centre distance between person *i* and *i* + 1, where *i* = 1, …, 9. The resulting time series and corresponding time average (dotted lines) are shown in Fig 11 for scenarios A–E. This demonstrates how one can account for physical distancing measures when simulating a queue.

**Fig 12:**
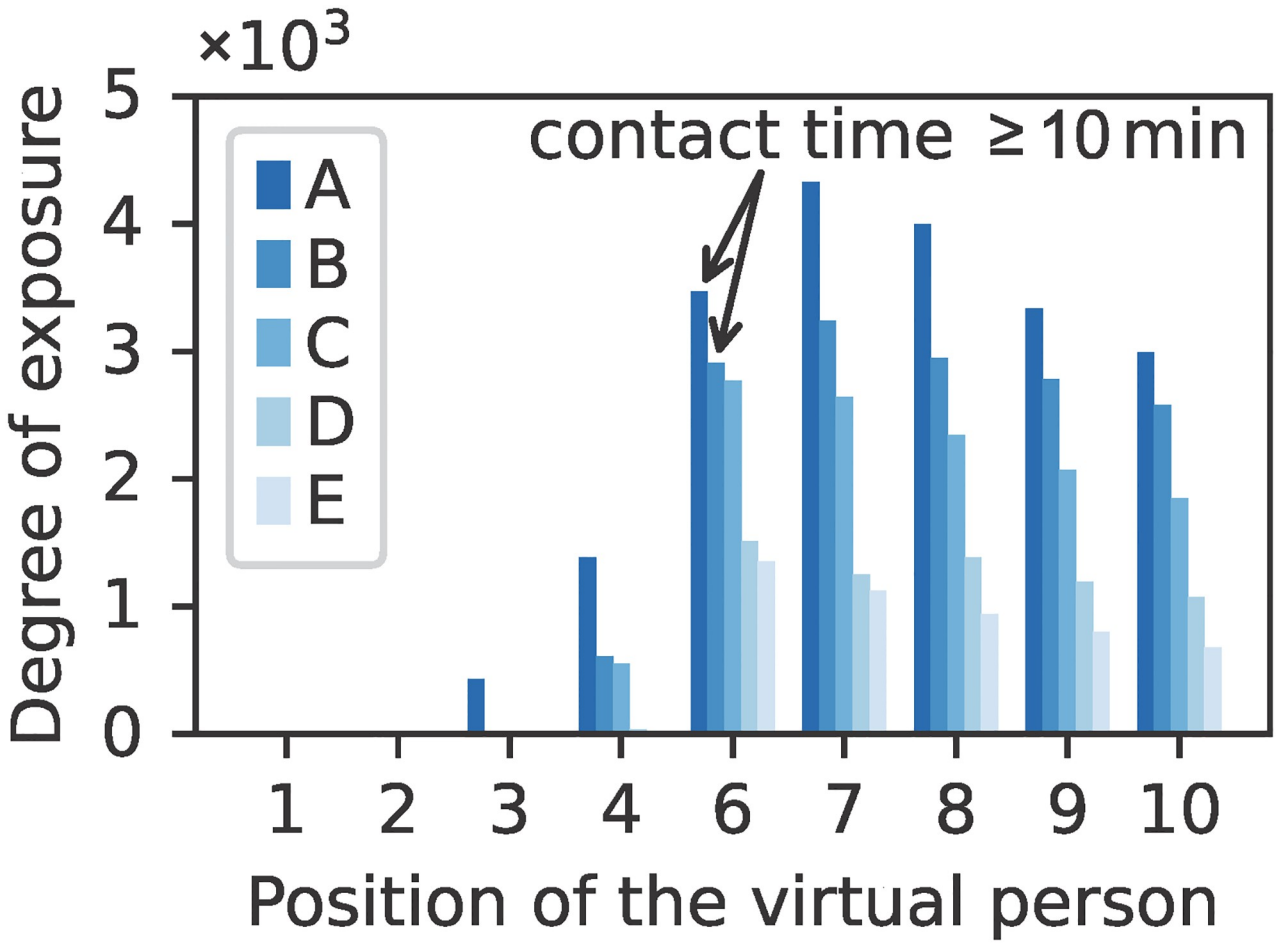
Individual degree of exposure for the queue scenarios. The variation of the potential parameter *p* and corridor distance *d*_*c*_ leads to a different exposure of the susceptible persons (1–4, 6–10). For scenario A and B, the contact time between the person at the sixth position and the infectious person, that is the period during which their mutual distance is less than 1.5 m, is greater than 10 min.

An increase in the mean distance affects the individuals’ degree of exposure, as can be concluded from Fig 12. Greater distancing between people $\bar{d_{p}}$ leads to less exposure. However, even in scenario E, we observe an exposure of over 10^3^ absorbed viruses, which could suffice for infection, particularly if a more infectious variant of the virus emerges. The average exposure of all virtual persons for scenario E decreases to 25% of the average exposure for scenario A.

### Discussion

In an exhaustive parameter study, one could take into account the uncertainty of other parameters. For example, we expect that modelling face masks or considering a less infectious person would reduce the shedding and intake of airborne pathogens, and thus the exposure, in the order of magnitudes.

A complementary sensitivity analysis, e. g. with variance-based methods, could be used to quantify the impact of input parameters and their interactions on the model output. We think that the parameter space of the transmission model is small enough so that such methods can be applied. However, the effort must not be underestimated since many parameters are likely to be influential. We plan to conduct a comprehensive sensitivity analysis and forward propagation, including the formal introduction of the mathematical theory, in a separate study because it would exceed the scope of the present work.

For now, our short parameter study of the queue scenario demonstrates the potential of applying uncertainty quantification methods. It shows that, measures such as physical distancing can help to reduce transmission of SARS-CoV-2. In this case, the protective effect is relatively low. It remains below one order of magnitude, even for scenario E in which the mutual distances are about 2 m. The measure might be costly because physical distancing occupies space, which is often limited in public areas. It is also difficult to adhere to because humans do not always estimate distances correctly.

In the introduction, we argued that exposure models should include long-range or time-dependent transmission because the airborne route is relevant for SARS-CoV-2. Our model takes these effects into account. Another key strength of the present study is that we use an established locomotion model that closely matches real pedestrian dynamics. The model can easily be parametrised to capture behavioural patterns such as social distancing. The combination of locomotion and transmission models enables us to evaluate the exposure in a queue with moving virtual persons. For such a scenario, other models that are based on proximity or exposure time fail to capture long-range transmission. In contrast to Wells-Riley-like models, we resolve the pathogen concentration spatially. Hence, we obtain an individual degree of exposure for each virtual person in the queue. The spatial resolution allows to analyse the effect of physical distancing on the individuals’ exposure. CFD simulations could reveal a more detailed picture of how the aerosols spread around the queue. Therefore, we will keep track of contributions from the CFD community that address not only the coupling of fluid dynamics and epidemiological modelling but also pedestrian dynamics.

Our simulation supports the claim that queues, in stagnant air, pose a severe exposure risk. However, how could this risk be reduced? We propose to evaluate measures by varying the model parameters that reflect these measures. For example, a mask worn by the infectious person would reduce the number of pathogens. In addition, physical distancing could be introduced. Organisers could strive to avoid this type of queue, e. g. by handing out service numbers, or they could install overhead ventilation.

## Conclusion and outlook

We complemented microscopic crowd simulation with a new model for the transmission of pathogens via small aerosol particles. The combined model was implemented in Vadere, an open-source framework for simulating pedestrian dynamics. Infectious persons exhale pathogens bound to aerosol clouds, whereas susceptible individuals absorb pathogens. We calibrated parameters to the transmission of SARS-CoV-2 and re-enacted two superspreading events for which we obtained qualitatively plausible results.

We demonstrated how to evaluate the risk of exposure in a typical everyday situation using our simulation model: We observed the number of pathogen particles absorbed by virtual persons queueing up in an unventilated room. We compared the results to a reference value obtained from a benchmark scenario, a close contact situation acknowledged as high risk in the context of SARS-CoV-2 by official health authorities. As long as there is no consensus on a dose-response model for SARS-CoV-2, we proposed interpreting virtual persons at high risk if they inhale as many viruses as in the reference scenario. A parameter study showed that, in a queue, several persons may reach a high-risk exposure although they are not close to the infectious person.

As a next step, we plan to refine the temporal and spatial spread of aerosols in the model. In addition, we propose to quantify uncertainties in the input parameters and in the quantities of interest, using sensitivity analysis and forward propagation. Thus, we hope to foster trust in the simulation results when we evaluate the effectiveness of measures or account for an evolving virus. Beyond that, we hope that our model will be adapted, when the need arises, by other scientists to investigate future pandemics.

## Supporting information

S1 Table Simulation seeds. All (pseudo-)random numbers used in the simulations with Vadere can be generated by using the listed seeds for the parameter fixedSeed.
(ZIP)

S2 Table Parameters of the Optimal Steps Model. We adapted the parameters of Vadere for the Optimal Steps Model to fit the virtual persons’ locomotion behaviour to the simulated situation.
(ZIP)

S1 Dataset Simulation data. The data sets contain the parameters and configurations for the simulation defined in the scenario file as well as the simulation outputs for all numerical experiments presented in this contribution. In addition, the scripts for the evaluation of the results are provided.
(ZIP)
