# Supplementary material for: Modelling airborne transmission of SARS-CoV-2 at a local scale
Source: PLoS One. 2022 Aug 30;17(8):e0273820. doi: 10.1371/journal.pone.0273820 (PMC9426895; doi:10.1371/journal.pone.0273820)
Supplement: S1 Table — All (pseudo-)random numbers used in the simulations with Vadere can be generated by using the listed seeds for the parameter fixedSeed. (ZIP) [file pone.0273820.s001.zip › S1_Table.pdf]

S1 Table. Simulation seeds.

**Scenario**	**Seed**
Close contact	8838372581797678424
Restaurant	4889043484410943750
Choir practice	−2054058476485033808
Queue	1436250873317888407

All (pseudo-)random numbers used in the simulations with Vadere can be generated by using the listed seeds for the parameter `fixedSeed`.
