# Supplementary material for: Modelling airborne transmission of SARS-CoV-2 at a local scale
Source: PLoS One. 2022 Aug 30;17(8):e0273820. doi: 10.1371/journal.pone.0273820 (PMC9426895; doi:10.1371/journal.pone.0273820)
Supplement: S2 Table — We adapted the parameters of Vadere for the Optimal Steps Model to fit the virtual persons’ locomotion behaviour to the simulated situation. (ZIP) [file pone.0273820.s002.zip › S2_Table.pdf]

S2 Table. Parameters of the Optimal Steps Model.

Scenario	seeSmallWalls	pedPotentialIntimateSpaceWidth	pedPotentialPersonalSpaceWidth	pedPotentialHeight	obstPotentialWidth	targetAttractionStrength
Default	false	0.45	1.2	50	0.8	1.0
Close contact	false	**0**	**0**	50	0.8	1.0
Restaurant	false	0.45	1.2	**5**	**0**	1.0
Choir rehearsal	false	0.45	1.2	**5**	0.8	1.0
Queue (1.0 m)	true	0.45	**0.5**, **1.0**	50	**0.4**	**0.1**
Queue (1.5 m)	true	0.45	**1.5**	50	**0.4**	**0.1**
Queue (2.0 m)	true	0.45	**2.0**, **2.5**	50	**0.4**	**0.1**

We adapted the parameters of Vadere for the Optimal Steps Model to fit the virtual persons' locomotion behaviour to the simulated situation.
